# Facilitating the initiation of the physician’s professional identity: Cornell’s urban semester program

**DOI:** 10.1007/s40037-014-0151-y

**Published:** 2014-11-14

**Authors:** Peter A. Goldstein, Carol Storey-Johnson, Sam Beck

**Affiliations:** 1 Department of Anesthesiology, Weill Cornell Medical College, 1300 York Avenue, Room A-1050, New York, NY 10065 USA; 2 Department of Medicine and the Office of Academic Affairs, Weill Cornell Medical College, New York, NY USA; 3 Urban Semester Program, College of Human Ecology, Cornell University, Ithaca, NY USA

**Keywords:** Role model, Mentor, Professionalism, College undergraduate, Moral development

## Abstract

Calling for major reform in medical education, the Carnegie Institute report ‘Educating Physicians’ espoused the importance of assisting student trainees in forming their professional identities. Here, we consider the question: At what educational stage should future physicians begin this process? The literature suggests that the process begins when students matriculate in medical school; we posit, however, that premedical students can begin their proto-professional development as college undergraduates. We describe here the pedagogy of Cornell University’s urban semester program (USP), which enables college students to participate in shadowing experiences as part of an integrated structured study programme. USP students report improved communicative competency, changes in their perceptions and attitudes toward medical practice, and powerful influences on their personal and professional development upon completion of the programme. We suggest the solution to the question of ‘When and under what conditions should shadowing take place?’ is to utilize a structure that combines the exposure of college students to the professional environment with a didactic and self-reflective curriculum, thereby supporting students in their early professional development. We conclude that educational efforts aimed at developing professional identity and behaviour can begin before students enter medical school.

## Introduction

‘Professional formation’ is a key requirement for medical student training [1], and is one of the six core competencies defined by the Accreditation Council for Graduate Medical Education. Medical role modelling, mentoring, and shadowing are the standard pedagogical devices that guide such development [1–6]. This kind of pedagogy is tied to Kolb’s ‘experiential learning’ (which combines experience, perception, cognition, and behaviour) [7] and the ‘hidden curriculum’ (‘a set of influences that function at the level of organizational structure and culture knowledge’) [8, 9] and, except for mentoring, is not perceived by the learner to be intentional as is the didactic, formal, and explicit curriculum. Critical medical practice values, attitudes and behaviours are learned in-context and in-process by imitating senior physicians and are reproduced over student generations. Medical professional identity formation, ‘the process one experiences during the transformation from lay person to physician,’ lies at the intersection of three overlapping domains—professionalism, psychosocial and cultural identity development, and formation [10]; it is a transformational process. Despite 100 years’ worth of reform in medical education, including efforts aimed at forging an appropriate professional identity in medical students as originally proposed by Flexner, professional formation remains an educational goal still in want of significant improvement [1].

The literature on professionalism and medical education [1, 11–14] indicates that the starting point of professional formation begins only when students matriculate in medical school (see Kitsis [15]). Early clinical experiences, in the first 2 years of medical school, have been shown to have numerous benefits [16], and may be useful in the early development of professional identity [10, 16]. We argue here that, because of the time required to develop a professional identity, medical educators are obliged to recognize that professional practice socialization and enculturation must start earlier than matriculation to medical school, at the pre-medical school level as students identify themselves as ‘pre-med.’ It is at this time that students should ‘shadow’ physicians, initiating medical professional formation, and reflect on ‘best’ versus ‘sub-optimal’ practices that they encounter in the clinical arenas. Kitsis criticized such shadowing by undergraduate college students due to concerns centring on fiduciary obligations, violations of privacy/confidentiality, potential for coercion, and misrepresentation [15]. The ethical concerns she raised have previously been addressed [17], and will not be further elaborated upon here. Kitsis concedes, however, that a ‘social obligation to educate future physicians exists,’ but believes that ‘this obligation begins [only] when students enter medical school’ [18], and it is this educational point that deserves further consideration.

The white paper *Medical Professionalism in the New Millennium: A Physician Charter* (put forth in 2002 by the ABIMF, ACP-ASIM, and EFIM^1^) stresses that medical professionalism is based on three principles: the primacy of patient welfare, patient autonomy, and social justice. It entails numerous responsibilities, including duty, advocacy, and ‘bedside manner’ among others. At the time the *Physician Charter* was published, and in accordance with the fundamental importance of the issue, the overwhelming majority of US medical schools offered formal curriculum content relating to professionalism and professional values [19]. Such efforts, however, have been less than successful at achieving the desired outcome [1, 20]. (and references therein). Importantly, the process of medical socialization and enculturation can, and does, begin well in advance of formal entry into medical school [9]. Thus, shadowing represents an excellent opportunity for college undergraduates to interact with role models-*cum*-mentors, thereby initiating the early development of an appropriate professional identity in proto-physicians.

### Closer look at the pedagogies

Shadowing is a form of experiential learning related to knowledge production methods used in internships, community service learning, and adult learning theory; it typically occurs in a one-to-one student–teacher setting in which the student is a passive observer [5]. It provides the student access to individuals who can serve as role models, mentors, or both. Notably, a role model is a ‘master’ who possesses expertise in knowledge and/or behaviour that a neophyte wishes to emulate but who may not be aware that he or she is an object to be emulated; in contrast, a mentor is typically an ‘experienced, highly regarded, empathetic person’ who intentionally ‘[guides] another (usually younger [and less experienced]) individual (the mentee) in the development and re-examination of their own ideas, learning, and personal and professional development’ [21]. Although the functions of a mentor may be different from that of a role model, such roles are not a priori mutually exclusive; indeed, role models may metamorphose into mentors. The education and learning literature has demonstrated that experiential learning may strengthen the participants’ sense of service, civic engagement, social justice, and increase their self-direction to influence social challenges [7, 22–24].

In medical education, role models, mentors, and imitating are fundamental to the acquisition of professional expertise and role performance, both of which are necessary components of professionalism [2–4, 25]. Due to the existence of a ‘hidden curriculum’ [9] and negative role models (which may be even more potent than positive ones) [26], however, role modelling and mentoring may be of limited value unless accompanied by guided structured reflection; as previously noted, ‘without a forum to share and reflect on their moral choices, students feel isolated and unable to resolve their identity and ethical conflict’ [1]. Unlike role models, who may be unaware of their role in the hidden curriculum, mentors intentionally teach. Over the course of 20 years, the Cornell University Urban Semester Program (USP) has worked with pre-medical school students who shadow Weill Cornell Medical College physicians in-context and in-process in hospital settings to initiate them to the culture and practice of medicine and professional practice. They come to understand aspects of hospital organizational culture, medical language and culture, and puzzle over behaviours they see in the course of shadowing. This experience combined with structured seminars requiring students to reflect on how doctors interact with patients, families, and other professionals, deepens their understanding of the clinical environment, medical culture, and professional practice.

## Methods

The USP is a course of study offered to Cornell University undergraduates who are interested in medical careers. The goals of the programme include: (1) demonstrating critical thinking skills; (2) identifying inequalities and disparities in health care environments; (3) identifying examples of inclusiveness and effective teamwork skills; (4) enhancing self-confidence and professional and personal self-identity development; and (5) identifying leadership skills. Students apply to gain entry to the programme. Each applicant is interviewed by the Programme Director to assess the student’s: (1) communicative ability, (2) assertiveness, (3) self-representation, and (4) desire for self-improvement.

Over the course of 20 years, the programme has established relationships with individual Weill Cornell Medical College departments and physicians, who self-select to mentor USP students, and create student shadowing rotations that are based on the students’ stated preferences when possible. In addition to shadowing rotations, students perform community service in North Brooklyn among low-income ethnically diverse populations and are asked to reflect on the relationship between the two experiential learning sites to explore differences and disparities in health care delivery and participate in each arena as participant observers.

Students initiate rotations by contacting faculty mentors in the speciality areas of medicine in which they are interested. After a brief interview, students are invited to participate in shadowing experiences. The activities in which students engage on the rotations vary, often including rounds on inpatient floors, observing physicians in the ambulatory, emergency, or operating arenas, and comforting or spending time with patients to understand their concerns.

Students average three to four different rotations over the course of the semester as ethnographers. They are encouraged to compare and contrast the different ‘cultures’ and behaviours that they encounter in different specialities. Their comments in seminars and in reflection journals revolve around what it means to be a ‘good doctor’ and how they imagine themselves as doctors in the future. A social and cultural anthropologist leads the seminars and creates the conditions for structured critical reflection. The USP students’ shadowing experiences are embedded in an integrative curriculum the goals and objectives of which are listed in Table 1.

**Table 1:** General goals, structure of, and activities in, the urban semester program

A. Programme goal: To enable advanced college students to engage medical culture and clinical professional formation
1. Integrative learning
i. Connect formal knowledge and problem-based reasoning skills to relevant clinical experience and skills
ii. Appreciate situated or in-context knowledge production located in sites of practice
iii. Develop insights into the skill levels necessary at different stages of educational development
iv. Experience organization culture of a hospital in relationship to the culture of medicine
v. Carry out immersion in communities of practice
2. Lifelong learning
i. Use inquiry and discovery to improve medical practice and professional formation
ii. Recognize that learning is progressive, developmental, dynamic, social, participatory, and distributed
iii. Recognize that learning is relentlessly reshaped, recombined, expanded and elaborated to improve the implementation of patient care
iv. Exercise self-directed learning through reflective practice
v. Use reflexivity to create self-awareness about assets and deficits to guide learning and self-improvement
3. Global learning
i. Use of an ethnographic/anthropological framework for generating knowledge
ii. Teamwork and the nature of collaboration across specialities and medical domains
iii. Improve the ability to communicate across inequalities and differences
iv. Deepen understanding of sociocultural and economic inequalities and their relationship to medical and health disparities
v. View patients as active participants in health improvement
vi. Comprehend the relationship of treating individuals but understanding population-based health issues
B. Structure: Weekly student seminars with physicians, researchers, and other health care professionals
Seminar #1: Physicians focus on issues related to:
i. Personal history of professional formation
ii. Decision-making process at different stages of professional formation
iii. Organization of medicine, from medical school admissions to health insurance coverage
iv. Advice about how to prepare for medical school
v. Disparities in health care and medical practice
Seminar #2: Ethnographic and experiential learning methods—readings and discussion
Seminar #3: Social medicine—readings and discussion
Seminar #4: Experiential learning reflections
Hospital rotations: NY-Presbyterian, Woodhull, Lincoln, NY-Methodist
Relationship of students with clinicians & researchers
Students are embedded into the normal routines of day-to-day activities, shadowing attendings, 3rd and 4th year medical students, physician assistants, and residents

## Results

What we sought to measure was the quality of the shadowing experience, rotation participation, reflection process, and personal and professional development in particular. In an anonymously completed end-of-course survey we queried USP students in general categories of learning that they experienced in the USP. We used a Likert scale with response options ranging from 1 (=very little) to 8 (=to a great extent) and we report their results at the high-end of the scale (responses 6–8). All course participants completed the survey. In a representative sample (n = 28), course participants were nearly equally divided by gender (13 males, 15 females), and were predominantly upper classmen (17 rising juniors, 7 rising seniors, 3 graduate students). We found that the medical shadowing, practice, and reflective sessions were seen as a powerful learning experience by 85 % of the participants. Nearly 80 % of USP students reported that participation in the programme expanded their understanding of the communicative processes involved in medical practice, resulting in an improved sense of communicative competency. Eighty-nine percent of the students reported that USP experiences influenced their personal development. In addition, 68 % indicated that the USP actually changed their perceptions and attitudes toward the practice of medicine. Finally, 86 % of participants said that their USP experiences influenced their professional development.

We also asked participants to provide illustrative comments on their learning experiences. As examples of the rich learning that occurs in the USP, the following comments were submitted by USP participants: ‘what is said is not usually what is done,’ (consistent with the existence of a ‘hidden curriculum’); regarding a paediatric patient who died, ‘although they [the doctors] showed her the love that every little girl should know in the world they were also able to shield themselves from the pain of her death’; and ‘to be a good doctor you can’t just be book smart and it really is crucial to have a certain composure and traits…’. Many such comments demonstrated an increased awareness that students gain about doctors and medical practice, contributing to their changing attitudes and perceptions.

## Discussion

While the Cornell USP is geared towards college students, it has many of the same qualities as the Leadership Opportunities with Communities, the Underserved, and Special Populations (LOCUS) programme offered at the University of Wisconsin Medical School (http://www.fammed.wisc.edu/medstudent/locus/what.html) [27], which has demonstrated success in helping medical school students develop leadership skills and maintain their ‘altruistic commitment to service’ [27]. Structurally, both Cornell’s Urban Semester and Wisconsin’s LOCUS programmes intrinsically respond to the implications inherent in Kohlberg’s moral development theory, which is that ‘*the major impetus for movement through the moral stages is the person’s own activity as problem solver, as called forth by challenging interactions with the environment*’ [28]. Shadowing is incorporated into both USP and LOCUS curricula, and it is a reasoned presumption that the students in each instance are interacting with physicians who are further along in their moral development and are perforce well-situated to act as role models-*cum*-mentors. Whether early exposure to physicians in their day-to-day work environment through participation in the USP leads to persistent changes in a student’s sense of what it means to be a medical professional remains to be determined; however, their comments reveal their capacity to adjust prior conceptual assumptions about the practice of medicine to better reflect the realities encountered in the clinical environment, a necessary transition for every professional student. This adjustment experience does not have to be experienced again when medical school is entered; it is an experience already completed and may be built upon.

The educational efforts aimed at facilitating the development of an appropriate sense of medical professionalism within the 4 year curriculum, while laudable, have to date been insufficient.[1]. Considering that the acquisition of the knowledge, skills, and wisdom necessary to be a professional does not occur overnight, we suggest that exposure to professional behaviour must occur early in a student’s education. Given the importance of professionalism in medicine, the need for experiential learning, and the necessity of exposure to appropriate role models and mentors in the early development of professional identity, we argue that the call for action to all medical and pre-medical school educators must be to start educational efforts aimed at developing professional identity and behaviour before, and not after, students enter medical school. Medical schools should strongly consider, collectively, and *in partnership with pre*-*medical school educational institutions*, enhancing and expediting the professional development of all future physicians by increasing opportunities for students to participate in programmes similar to Cornell’s Urban Semester Program.

## Footnotes

1 ABIMF: American Board of Internal Medicine Foundation, ACP–ASIM: American College of Physicians–American Society of Internal Medicine, EFIM: European Federation of Internal Medicine.
